# Fibroepithelial Polyp of the Ureter: A Rare Cause of Hydronephrosis

**DOI:** 10.1089/cren.2018.0031

**Published:** 2018-10-01

**Authors:** Murat Uçar, Ercan Baş, Ali Akkoç, Murat Topçuoğlu

**Affiliations:** ^1^ Department of Urology, Alanya Alaaddin Keykubat University School of Medicine, Antalya, Turkey.; ^2^ Department of Urology, Suleyman Demirel University School of Medicine, Isparta, Turkey.

**Keywords:** ureter, polyp, ureterorenoscopy, cauterization

## Abstract

***Background:*** Fibroepithelial polyps of the urothelial system are rare and are considered to be benign tumors. Ultrasonography (USG), contrast-enhanced CT, and contrast-enhanced MRI can be used for detecting fibroepithelial polyps in the urothelial system. These polyps can be treated by performing open exploration and endoscopic or laparoscopic resection. Previous studies have also reported the frequent use of laser treatment for treating fibroepithelial polyps located in the proximal ureter.

***Case Presentation:*** A 54-year-old female patient presented to our clinic with right flank pain. Evaluation of the patient by performing USG and CT detected grade-2 hydronephrosis of the right kidney; however, no stone was detected in the urinary system. MRI detected thickening of the wall of the right proximal ureter along with contrast enhancement. These findings suggested the presence of a ureteral polyp. Ureterorenoscopy detected a 7-cm-long ureteral polyp in the proximal ureter, which was resected by performing monopolar cautery.

***Conclusion:*** Although fibroepithelial polyps of the urinary tract are rare, they should be considered in the absence of urinary calculi and in the presence of a ureteral obstruction. Furthermore, careful endoscopic resection by performing electrocautery is a safe and useful method for treating ureteral lesions.

## Introduction

The incidence of fibroepithelial polyps in urinary collecting system is 2% to 6%.^1^ These polyps are benign tumors and originate from the mesoderm. Although fibroepithelial polyps are rare, they are the most common benign tumors of the ureter.^2^ It is important to distinguish between fibroepithelial polyps and urothelial carcinoma because these two conditions require different management strategies and show different prognosis. Although open exploration and resection have been used previously for treating fibroepithelial polyps, less invasive approaches are used at present. In this study, we report the case of a patient with a fibroepithelial polyp in the proximal ureter that was treated by performing resection with ureterorenoscopy.

## Case

**FIG. 1.**
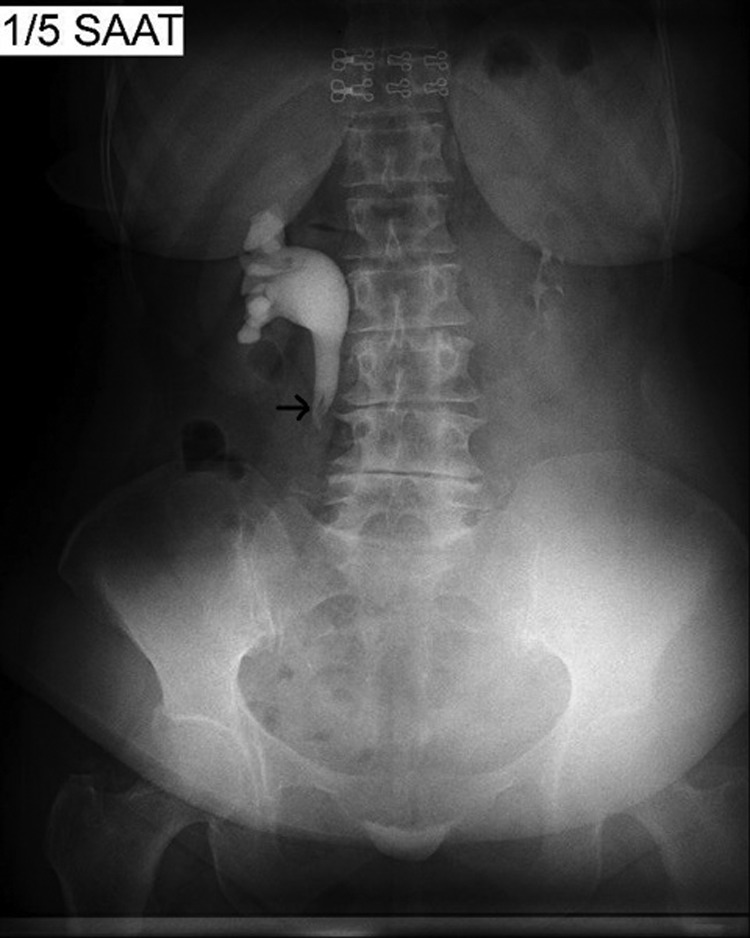
İntravenous pyelogram showing filling defects (*arrow*) in right proximal ureter corresponding to fibroepithelial polyps. Distal ureter is not still observed at fifth hour after administration of contrast material.

**FIG. 2.**
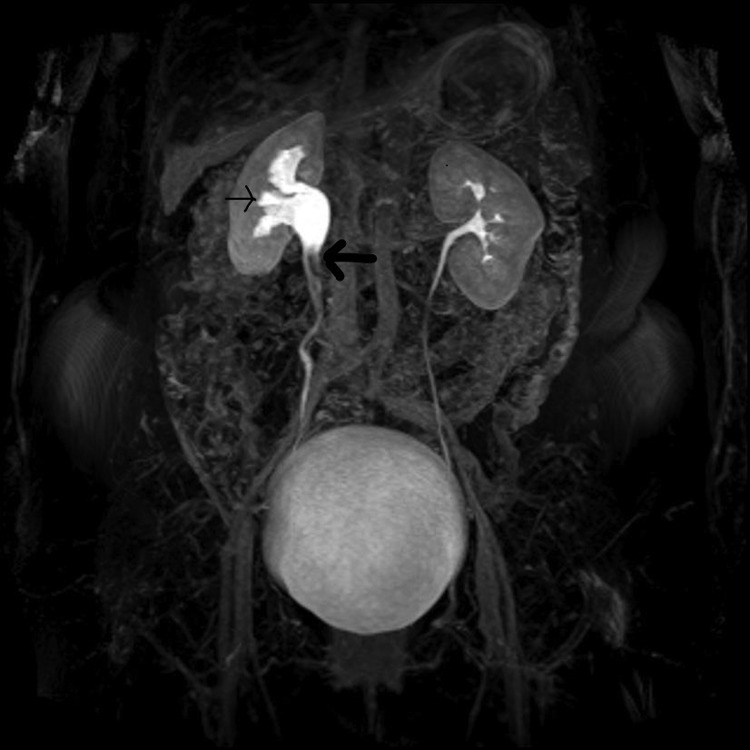
Three-dimensional MR urography with T2-weighted images; filling defects in right proximal ureter (*bold arrow*), causing ureteral obstruction and moderate pyelocaliceal upstream dilation (*thin arrow*).

**FIG. 3.**
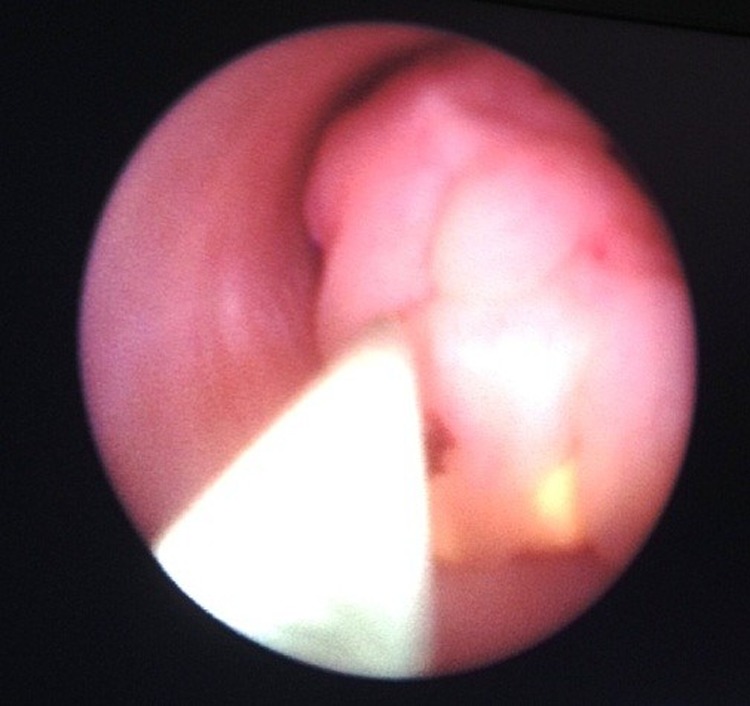
Ureterorenoscopic image of fibroepithelial polyp.

**FIG. 4.**
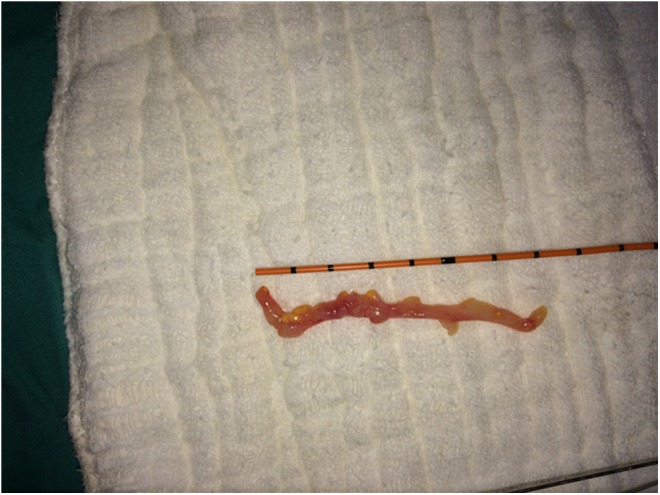
Macroscopic demonstration of fibroepithelial polyp.

**FIG. 5.**
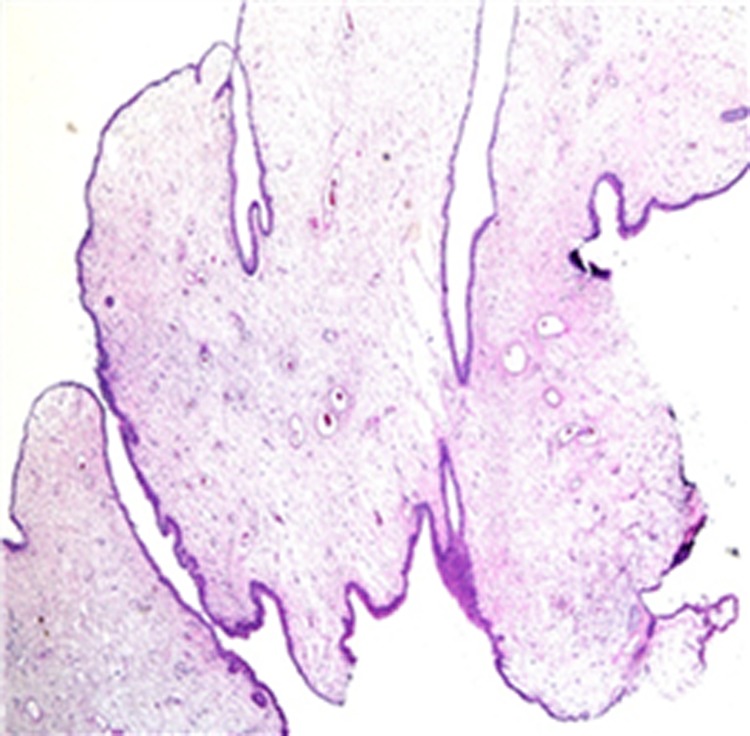
Histologic preparation of fibroepithelial polyp showing stroma covered by layer of normal transitional epithelial cells (Hematoxylin&Eosin 40 × ).

A 54-year-old female patient presented with colic right flank pain. She did not have any urinary tract infection, stone, or tumor in her history. Urinalysis did not detect microscopic hematuria or pyuria. Moreover, her renal functions were normal, and urine culture did not yield any abnormal findings. Ultrasonography (USG) detected grade-2 hydronephrosis of the right kidney. Noncontrast CT did not detect any stone in the urinary system. However, intravenous urography (IVU) detected filling defects in the right proximal ureter (Fig. 1). MRI detected thickening of the wall of the right proximal ureter along with contrast enhancement (Fig. 2). These findings suggested the presence of a fibroepithelial polyp in the right proximal ureter. Ureterorenoscopy detected a 7-cm-long stemmed lesion originating from the right proximal ureter (Fig. 3). The lesion was resected by performing monopolar cautery with a Bugbee electrode, and the specimen was extracted through the ureteral orifice and was exteriorized outside the urethra by using forceps (Fig. 4). After the resection, the stalk of the polyp was cauterized. Resection of the polyp and cauterization of the ureteral wall was performed carefully to prevent ureteral perforation. Next, a 4.8F Double-J ureteral stent was placed in the ureter for 2 weeks. Histopathological analysis of the lesion indicated that it was a fibroepithelial polyp with negative surgical margins (Fig. 5). The patient was discharged on the first postoperative day and has had an uncomplicated postoperative course thus far.

## Discussion

A ureteral fibroepithelial polyp was first reported in 1932. Fibroepithelial polyps most commonly develop in adults and children. Moreover, their incidence is more common in men in the third or fourth decade of life than in women.^1,2^ These polyps contain stroma derived from the mesoderm and are covered by a layer of normal transitional epithelial cells. Although the etiology of these tumors has not yet been clearly understood, it is suggested to be associated with congenital, irritative, infectious, obstructive, and traumatic causes. The most frequent clinical findings of patients with fibroepithelial polyps are flank pain and hematuria. Moreover, these patients may present with less common findings such as urinary frequency, dysuria, or pyuria. The first diagnostic evaluation of all patients should include IVU, contrast-enhanced CT, or contrast-enhanced MRI. Determination of filling defects by performing antegrade or retrograde pyelography may also help in diagnosis. Clinical symptoms and results of imaging tests are inadequate for the definitive diagnosis of fibroepithelial polyps. The definitive diagnosis of fibroepithelial polyps can be established by performing histopathological analysis. Biopsy or resection by using open, endoscopic, or laparoscopic techniques should be performed to obtain specimens for performing histopathological analysis of fibroepithelial polyps. Debruyne et al. reported that 41 (37%) unnecessary nephrectomies were performed in 108 patients because of an uncertain preoperative diagnosis.^3^ Ureterorenoscopy is also useful for differentiating between benign and malignant ureteral lesions.

Fibroepithelial polyps typically present as smooth, mobile, and pedunculated masses in the ureter.^1^ Franco et al. reported that none of the patients with fibroepithelial polyps showed malignant transformation.^4^ In some patients, fibroepithelial polyps may be accompanied with transitional cell carcinoma. Moreover, fibroepithelial polyps may develop in different locations in the urinary system, including the ureter, renal pelvis, bladder, and urethra.

To date, no study has compared the efficacy of holmium laser treatment with that of electrocautery. Electrocautery may damage the ureter and surrounding tissues during tumor resection and cauterization. Moreover, electrocautery may result in perforation, adhesion, stenosis, and fistula formation in the ureter after tumor resection. Therefore, electrocautery should be performed carefully to prevent these adverse events. Our patient was treated by performing electrocautery because of the unavailability of laser treatment in our clinic at that time.

Previously, fibroepithelial polyps were treated by performing open exploration and resection. At present, with developments in endoscopic and laparoscopic techniques and tools, patients with fibroepithelial polyps can be treated endoscopically, percutaneously, or laparoscopically. Percutaneous antegrade approach is preferred for the ablation of polyps located in the renal pelvis or proximal ureter. Ho-YAG laser treatment or endoscopic cauterization is preferred for treating polyps in the distal ureter that can be reached through a retrograde approach. At present, laser treatment is performed more frequently for treating fibroepithelial polyps located in the proximal ureteral. In our case, the polyp in the proximal ureter was resected by performing electrocautery with ureterorenoscopy. Laparoscopy has been performed to resect large and elongated polyps and is associated with some advantages such as improved visualization and easy working space. Endoscopic resection can be performed safely in patients with small papillary lesions in the ureter. Ureterorenoscopy is a widely used, less invasive, and cost-effective alternative to open and laparoscopic surgery. It is recommended to leave a Double-J stent in the ureter after resection. Ureteral Double-J stents are usually left in the ureter for ∼6 weeks. In our case, the Double-J stent was left in the ureter for 2 weeks after the resection, which is different from that recommended in the literature.

In conclusion, although fibroepithelial polyps of the urinary tract are rare, they should be considered in the absence of urinary calculi and in the presence of a ureteral obstruction. Moreover, diagnostic and curative ureterorenoscopy should be performed in patients suspected of having fibroepithelial polyps. In our case, the fibroepithelial polyp located in the proximal ureter was resected by performing endoscopic resection. We believe that careful endoscopic resection by using a laser or cautery can be used for treating ureteral lesions.

## Abbreviations Used

CT: computer tomography
IVU: intravenous urography
MRI: magnetic resonance imaging
USG: ultrasonography
